# The collagenase-1 (MMP-1) gene promoter polymorphism - 1607/2G is associated with favourable prognosis in patients with colorectal cancer

**DOI:** 10.1038/sj.bjc.6603630

**Published:** 2007-02-20

**Authors:** A Hettiaratchi, N J Hawkins, G McKenzie, R L Ward, J E Hunt, D Wakefield, N Di Girolamo

**Affiliations:** 1 Inflammatory Diseases Research Unit, Department of Pathology, School of Medical Sciences, University of New South Wales, Sydney, Australia; 2 Histology and Microscopy Unit, School of Medical Sciences, University of New South Wales, Sydney, Australia; 3 Department of Medical Oncology, St Vincent's Hospital, Sydney, Australia

**Keywords:** metalloproteinase, real-time polymerase chain reaction, single nucleotide polymorphism

## Abstract

Matrix metalloproteinase (MMP) overexpression has been implicated in the pathogenesis of colorectal carcinoma (CRC). Accumulating evidence suggests that MMP promoter single nucleotide polymorphisms (SNPs) effecting gene transcription are associated with enhanced susceptibility for the development of malignant disease, increased tumour invasiveness and poor patient survival. The aim of the current investigation was to determine whether such associations exist in a large CRC patient/control study population. Using an allelic discrimination real-time polymerase chain reaction, polymorphisms in the MMP-1, MMP-2 and MMP-3 gene promoters (−1607, −1306, and −1612 bp, respectively) were assessed in normal blood mononuclear cells from patients with CRC (*n*=503) and control subjects (*n*=471). Genotypes corresponding to each MMP SNP were correlated with tumour characteristics and clinical outcome. The frequency of each genotype was not statistically different between patients and control subjects and no significant differences were noted between the genotypes and tumour characteristics for the three MMP SNPs. CRC patients with the 2G/2G genotype for the MMP-1 SNP had significantly better 5-year survival compared to patients with a 1G allele (*P*<0.05). Our results demonstrate that CRC patients with a 2G/2G genotype in the MMP-1 gene promoter SNP have a favourable prognosis. Although our results were unexpected, given that this genotype is associated with enhanced MMP-1 transcriptional activity, they are consistent with recent data highlighting the anti-tumorigenic properties of MMPs.

Colorectal carcinoma (CRC) is one of the most common malignancies in the western world. Despite advances in surgical techniques, improved chemotherapy, and early detection, this malignancy is still associated with a relatively poor prognosis and at least 40% of patients who undergo resection of the primary tumour die within 5 years either because of local recurrence or metastatic disease (Midgley and Kerr, 1999). Currently, the most clinically useful prognostic indicator in CRC is tumour stage, as determined by the extent of local tumour invasion and metastatic spread. Degradation of extracellular matrix is required for tumour cell migration and dissemination, a process that is facilitated by a family of neutral proteolytic enzymes known as the matrix metalloproteinases (MMPs) (Wagenaar-Miller *et al*, 2004). Given their critical role in cancer, attempts have been made to design synthetic MMP inhibitors (MMPI) in the hope of limiting metastatic spread. Results from clinical trials using MMPIs have been disappointing, predominantly because of severe side effects as well as the lack of tumour responsiveness to these agents (Coussens *et al*, 2002).

Matrix metalloproteinases are a family of calcium- and zinc-dependent endopeptidases that share amino-acid sequences, structural domains, and overlapping substrates. These enzymes are secreted as zymogens and removal of an activation peptide is required for their proteolytic activity (Nagase and Murphy, 2004). Matrix metalloproteinases maintain and remodel tissue architecture under normal physiological and pathological conditions (Nagase and Murphy, 2004). They are broadly divided into the collagenases, gelatinases, stromelysins, and membrane-associated-MMPs on the basis of their substrate specificity. Overexpression of MMP-1 (Murray *et al*, 1996), MMP-2 (Papadopoulou *et al*, 2001), and MMP-3 (Roeb *et al*, 2004) has been linked to poor prognosis, high tumour stage, and enhanced tumour invasiveness in patients with CRC. These subjective and semiquantitative studies have inspired subsequent investigators to assess genetic polymorphisms in the promoter regions of MMP genes, as this type of DNA sequence alteration can affect cancer susceptibility and malignant phenotype (Yasui *et al*, 2005).

In relation to tumour progression, MMP-1 has received relatively little attention compared to the basement membrane-degrading proteinases (MMP-2, -9). The promoter region of the MMP-1 gene contains consensus sequences for DNA-binding proteins such as AP-1, AP-2, Ets/PEA-3, and responsive elements to glucocorticoids, retinoic acid, and cyclic AMP (Rutter *et al*, 1997). A single nucleotide polymorphism (SNP) has been identified at position −1607 bp within the MMP-1 promoter region, whereby the insertion of an additional guanine (G) residue creates an extra Ets-binding site (Rutter *et al*, 1998). A promoter containing this SNP (giving rise to the 2G genotype) displays significantly ‘higher’ transcriptional activity in normal and malignant cells compared to cells possessing a 1G allele (Rutter *et al*, 1998; Wyatt *et al*, 2002) with ‘lower’ transcriptional activity. Several studies have identified strong linkage between this polymorphism and several common malignancies, including colorectal (Ghilardi *et al*, 2001; Hinoda *et al*, 2002; Zinzindohoue *et al*, 2005; Elander *et al*, 2006), endometrial (Nishioka *et al*, 2000), lung (Zhu *et al*, 2001), and ovarian (Kanamori *et al*, 1999) cancers and melanoma (Rutter *et al*, 1998; Tower *et al*, 2002). Polymorphisms in the promoter of MMP-2 (Miao *et al*, 2003), MMP-3 (Ghilardi *et al*, 2002), and MMP-7 (Zhang *et al*, 2005) have also been associated with increased susceptibility to the development of cancer, and to poorer prognosis, but these associations have not been found in all studies (Lei *et al*, 2002; Wenham *et al*, 2003; Fong *et al*, 2004; Krippl *et al*, 2004; Zinzindohoue *et al*, 2005).

We undertook the current investigation to determine whether an association could be found between the MMP-1, -2, and -3 gene promoter SNPs and CRC using the largest patient/control cohort to date. A reliable, rapid, non-radioactive real-time PCR method was developed to enable high sample throughput. Our results demonstrate that of the SNPs studied, none were associated with increased susceptibility to developing CRC, nor was there a significant correlation with any tumour characteristic analysed. Surprisingly, and contrary to other reports, we found that patients with CRC carrying the MMP-1 2G SNP had significantly improved 5-year survival. These findings may be of significance given the recent development and clinical trialing of MMP inhibitors.

## MATERIALS AND METHODS

### Study subjects

This study included 503 CRC patients without synchronous metastases who had undergone curative resection at St Vincent's Hospital, Sydney (Table 1). Enrolment of CRC patients in the study was between 1 January 1994 and 29 May 2004, and patient follow-up was performed for a period of up to 5 years or until death. Cancer recurrence dates and causes of death were obtained from medical records and death certificates where appropriate. Following informed consent, peripheral blood samples were obtained from all patients. This aspect of the study was approved by the St Vincent's Hospital Human Research Ethics Committee. Histological analysis of the tumours has been described previously (Ward *et al*, 2003). Peripheral blood from 471 healthy controls was obtained from volunteer donors at the Red Cross Blood Bank, Sydney, over a 3-week period after they provided informed consent for their DNA to be used anonymously for research into genetic predisposition to CRC. This aspect of the study was approved by the Red Cross Blood Bank Human Ethics Committee. For nucleotide sequencing, genomic DNA (gDNA) was extracted from peripheral blood mononuclear cells and amplified by PCR and real-time PCR using standard methods. Tumour-derived gDNA was randomly selected from 61 CRC patients known to be heterozygous for the MMP-1 polymorphism and used to study loss of heterzygosity (LOH).

The clinical staging of all tumours was completed according to AJCC/UICC guidelines. The anatomical location of the tumour was categorised into two groups: right-sided (proximal to and including the splenic flexure) or left-sided (descending colon, sigmoid, or rectum). Tumour margin type was established as described by Jass *et al* (1987); circumscribed when the margin was pushing or reasonably well circumscribed, or as infiltrating when the tumour invades in a diffuse manner with widespread penetration of normal tissues. The level of mucin production was determined as positive if present in >50% of the tumour area. Intra-epithelial lymphocytes were classified as inconspicuous (⩽20 mm^−2^) or prominent (>20 mm^−2^).

### Amplification of DNA for nucleotide sequencing

Polymerase chain reaction (PCR) was carried out on gDNA using specific forward and reverse primer pairs (Sigma Genosys, Australia) designed to span the MMP-1 (5′-TTATGCCACTTAGATGAGGAAATTGT-3′ and 5′-CACTTTCCTCCCCTTATGGATTC-3′), MMP-2 (5′-TCTGGGCCATTGTCAATGTTC-3′ and 5′-TCAAGGAAGGCTTCCTGGAA-3′) and MMP-3 (5′-CGGCACCTGGCCTAAAGAC-3′ and 5′-TCCTCATATCAATGTGGCCAAA-3′) polymorphic sites to generate PCR products of 151/ 152, 117, and 127/ 128, respectively. Reactions were carried out in a final volume of 25 *μ*l, containing 50 ng of gDNA, 15 pmol of each primer, 10 mM of each dNTP, 20 mM Tris–HCl (pH 8.4), 50 mM KCl, 1 mM MgCl_2_ and 1 U Platinum *Taq* DNA polymerase (Invitrogen, Carlsbad, CA, USA). Thermal cycling was performed in a GeneAmp PCR System 2400 thermal cycler (Perkin Elmer, Foster City, CA, USA) using the following conditions: 95°C for 1 min, followed by 30 cycles of amplification (95°C denaturation for 30 s, 55°C annealing for 30 s, 72°C extension for 30 s), and a final extension at 72°C for 5 min. Amplified products were separated on a 2% agarose gel, stained with 5–10 *μ*g ml^−1^ ethidium bromide and visualised under UV light. PCR products were gel-purified using the Wizard SV Gel and PCR Clean-Up System kit (Promega Co, Madison, WI, USA) and their nucleotide sequence determined by a commercial sequencing facility (Sydney University and Prince Alfred Molecular Analysis Centre (SUPAMAC)).

### Real-time PCR allelic discrimination analysis

Matrix metalloproteinase-1, MMP-2 and MMP-3 gene promoter SNPs were analysed by a real-time PCR allelic discrimination TaqMan assay (Applied Biosystems Inc., Foster City, CA, USA) on an ABI Prism 7700 Sequence Detection System (Applied Biosystems Inc.). The primers and TaqMan minor groove binding (MGB) probes for the MMP-1 (1G allele 6FAM-AGTTAAATAATTAGAAAGATATGACTTATC-NFQ and 2G allele VIC-AGTTAAATAATTAGAAAGGATATGACTTATC-NFQ), MMP-2 (C allele 6FAM-AGCACTCCACCTCTTTAGCT-NFQ and T allele VIC-AGCACTCTACCTCTTTAGCT-NFQ) and MMP-3 (5A allele 6FAM-GGGAAAAACCATGTCTTGT-NFQ and 6A allele VIC-GGGAAAAAACCATGTCTTGT-NFQ) were designed using Primer Express v2.0 software (Applied Biosystems Inc.), and the MGB probes were synthesised by Applied Biosystems Inc. Primer specificity was examined under standard PCR conditions before use in the allelic discrimination assay. Reactions were run in duplicate with the appropriate no template (NTC) and/or no amplification (NAC) controls. Each reaction consisted of 1 × Rainbow Probe Master Mix (Quantace Ltd, Watford, UK), 500 nmol l^−1^ each primer, 100 nmol l^−1^ each TaqMan MGB probe and 50 ng template in a final volume of 20 *μ*l. Thermal cycler conditions were, 50°C for 2 min, 95°C for 10 min followed by 35 cycles of 92°C for 15 s and 60°C for 1 min. All real-time PCRs were monitored and analysed using the Sequence Detector v1.7 software (Applied Biosystems Inc.).

### Immunohistochemical analysis of tissue microarrays

Paraffin-embedded CRC blocks were obtained from St Vincent's Hospital, Sydney. Haematoxylin and eosin stained slides from all cases were reviewed and the tumour tissue was selected and marked. Tissue micro arrays (TMAs) containing two 1-mm-diameter cores from each tumour were constructed using a manual arrayer (Beecher Instruments, Silver Spring, MD, USA). From each of the resulting 12 arrays, 4 *μ*m sections were immunostained with an MMP-1 monoclonal antibody (Calbiochem, Darmstadt, Germany, clone 41–1E5) at a 1 : 600 dilution for 30 min on a Bond X™ automated immunostainer (Vision BioSystems, Melbourne, Australia) using a Polymer Defined Detection System, Vision Biosystems, Melbourne, Australia. Sections were counterstained with haematoxylin and a staining index developed to report the intensity of MMP-1 positivity within the epithelial component of the TMA, with 3+ equivalent to high, 2+ indicating medium, 1+ corresponding to low, and 0 indicating absence of MMP-1 staining. Interestingly, MMP-1 staining was either present or absent within the stromal component of diseased tissue and was scored appropriately. The immunostained TMAs were reviewed by three independent observers who were masked to the MMP-1 genotype of each patient. Discordant observations were resolved by attaining consensus, and when this could not be achieved, the core was omitted from the analysis (*n*=11, ∼3%). Negative controls include sections incubated without a primary antibody or in the presence of an isotype-matched control antibody for which no reactivity was observed.

### Statistical analyses

Statistical analyses were performed using SPSS 13.0. software package for Windows (SPSS Inc., Chicago, IL, USA). The *χ*^2^ test was used to compare differences in categorical variables between the CRC patients and the control group, as well as within the CRC patient group for each of the MMP SNP sites. Cox regression analysis was used to calculate the odds ratio (OR) and 95% confidence interval (CI) to test the association between MMP promoter genotype and the risk of CRC. Survival times were measured from the date of resection to the date of death, and cases were censored at the completion of 5 years of follow-up, or at the last clinical visit before 30 June 2005. Non-cancer-related deaths were censored at the date of death. The log rank test was used to test the statistical difference between Kaplan–Meier survival curves (Kaplan and Meier, 1958). The threshold for significance was *P*<0.05.

## RESULTS

### Patient demographics and MMP allelotype distribution

The study population (Table 1) consisted of 503 patients with CRC (265 (52.4%) males and 238 (47.3%) females) with an age range of 28.6–99 years, as well as 471 control subjects with an age range of 17.6–78.6 (281 (59.7%) males and 190 (40.3%) females). Patients and control subjects were derived from the same geographic location and are representative of an Australian population. The gender distribution between patients and control subjects was not statistically significant (*P*=0.13). The genotype distribution for MMP-1 (1G and 2G) did not deviate significantly (*P*=0.78) between patients with CRC (51.0 and 49.0%, respectively) and controls (50.4 and 49.6%, respectively). In addition, there was no significant difference recorded between patients and controls for the MMP-2 (*P*=0.61) or the MMP-3 (*P*=0.21) allelotype frequencies (data not shown).

### Matrix metalloproteinase genotype distribution and susceptibility to CRC

**Figure 1:**
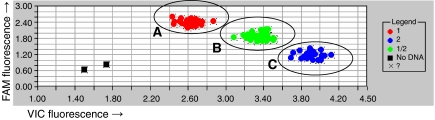
Matrix metalloproteinase-1 allelic discrimination assay plot. A representative genotype assay plot to demonstrate segregation of the three possible genotypes generated for MMP-1 SNP (circle A, 1G/1G homozygotes; circle B, 1G/2G heterozygotes; circle C, 2G/2G homozygotes) based on fluorescence intensity (x axis, VIC fluorescence; y axis, FAM fluorescence) of each PCR product. For quality control reassurance, sequenced samples for each genotype were amplified in each assay run along with no template controls (black squares, bottom left corner of plot) for which no products formed. Similar plots were generated for the MMP-2 and MMP-3 gene promoter SNP (data not shown).

**Figure 2:**
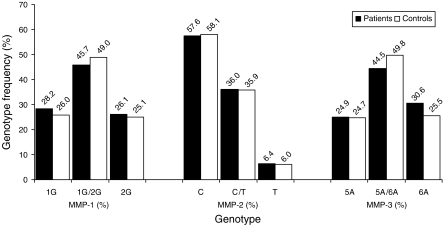
Distribution of the MMP-1, -2, and -3 gene promoter polymorphism in the study population. Genotype frequency for each MMP promoter polymorphism was established from the allelic discrimination PCR-based assay. Each genotype is expressed as a percentage of the total number of patients (black bars) or control subjects (white bars). No significant deviation in the distribution of each genotype for any of the three MMP polymorphisms was noted between patients with CRC compared to control subjects.

Each MMP gene promoter SNP was determined by an allelic discrimination real-time PCR assay. Typical dot plots were generated to illustrate the distribution of MMP-1 (Figure 1), MMP-2 and MMP-3 (data not shown) promoter polymorphisms in our study population. Patients and control subjects were segregated according to genotype (Figure 1: A 1G/1G (1G homozygous); B, 1G/2G (heterozygous); C, 2G/2G (2G homozygous) based on fluorescence intensity. Each assay was calibrated by incorporating control gDNA from subjects that had sequence proven polymorphisms of the MMP-1, -2, -3 promoters. Furthermore, the allelic discrimination PCR assay was validated by sequencing 90 patients with CRC (10 patients of each genotype (7.5% of the total study population) for each of the MMP polymorphic sites and the results showed 100% concordance (data not shown). The MMP-1, -2, and -3 promoter genotypes were successfully determined for 490 (97.4%), 486 (96.6%), and 470 (93.4%) of the CRC patients and 470 (99.9%), 467 (99.1%), and 452 (96.0%) of the control subjects, respectively. There was no significant difference in the genotype distribution (*P*>0.05) for the three MMP SNPs examined between patients and controls (Figure 2). The discrepancy between the number of subjects enrolled in the study and the actual number genotyped reflected failure to generate a sequence profile for some subjects. It is likely that a PCR inhibiting contaminant within the failed gDNA samples resulted in our inability to obtain a genotype call from either the real time assay or from sequence analysis. In any case, this failure rate was <3% in both CRC cases and controls for the MMP-1 SNP.

### Matrix metalloproteinase genotype and clinical phenotype

Potential associations were explored between MMP genotype and phenotypic tumour characteristics such as tumour stage, differentiation, TMN resection status, mucinous phenotype, lymphatic, vascular, or perineural invasion. No significant differences were detected between these and other tumour characteristics and MMP genotypes, irrespective of whether the analysis was performed as ‘high’ *vs* ‘low’ (viz., 2G/2G genotype *vs* both 1G/2G and 1G/1G, Table 2) or ‘low’ *vs* ‘high’ (viz., 1G/1G genotype *vs* both 1G/2G and 2G/2G) MMP-1 expression (data not shown).

### Matrix metalloproteinase genotype and survival analysis

**Figure 3:**
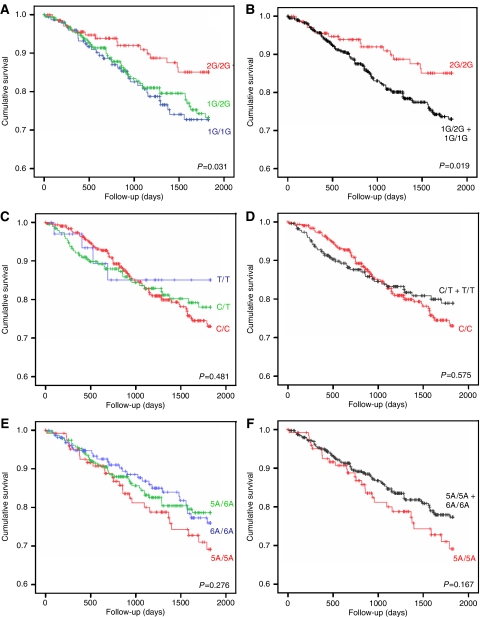
Kaplan–Meier curves for overall survival stratified by MMP promoter genotype. Survival curves for the MMP-1 (**A** and **B**), MMP-2 (**C** and **D**), and MMP-3 (**E** and **F**) gene promoter polymorphisms were generated based on individual genotype (**A**, **C**, **E**) or grouped on the basis of high MMP expression (one genotype) compared to low MMP expression (two genotypes) (**B**, **D**, **F**). No significant difference in survival was noted for the MMP-2 or MMP-3 SNP, irrespective of the method of analysis (*P*>0.05). However, patients homozygous for the 2G allele in the MMP-1 promoter (one genotype analysis) had a favourable prognosis (*P*=0.031) when compared to patients with a 1G allele (**A**). When the genotypes associated with low MMP-1 expression (1G/1G and 1G/2G) were combined and compared to high MMP-1 expression (2G/2G), patient survival was further enhanced (*P*=0.019).

Correlation between the MMP-1, -2, and -3 genotypes and survival was analysed and plotted using Kaplan–Meier survival curves (Figure 3). Patient survival was analysed on the basis of a single genotype or stratified according to ‘high’ MMP expression (one genotype) compared to ‘low’ MMP expression (two genotypes). Patients homozygous for the MMP-1 1G allele exhibited significantly poorer survival compared to those carrying a 2G allele (*P*=0.031). The Kaplan–Meier curves generated for this polymorphism demonstrated a pattern suggestive of a dose–response effect (Figure 3A). Likewise, when the MMP-1 genotypes were analysed according to ‘high’ (2G/2G) compared to ‘low’ (1G/1G plus 1G/2G) MMP-1 expression, patients homozygous for the 2G allele had significantly improved survival rates (*P*=0.019) (Figure 3B). In contrast, no significant association was found between the MMP-2 and MMP-3 genotypes and survival in patients, irrespective of the genotype grouping utilised, for example as a single genotype (Figure 3C and 3E) or a ‘high’ (C/C) *vs* combined ‘low’ (C/T and T/T) MMP-2 expression (Figure 3D), or ‘high’ (5A/5A) *vs* combined ‘low’ (5A/6A and 6A/6A) MMP-3 expression (Figure 3F).

### Multivariate analysis

A multivariate analysis (Cox regression) was performed for each polymorphism using two different genotype groupings (Table 3). It was found that the OR of CRC-associated death for the MMP-1 2G/2G homozygous genotype compared with the 1G/2G and 1G/1G genotypes was 0.427 (95% CI 0.19–0.96, *P*=0.04). The distribution of the other genotypes relating to MMP-2 and MMP-3 showed no significant difference (OR 1.16; 95% CI 0.66–2.04, OR 1.59; 95% CI 0.87–2.84, respectively) with respect to CRC-related death (Table 3).

### Immunohistochemical analysis of TMAs

**Figure 4:**
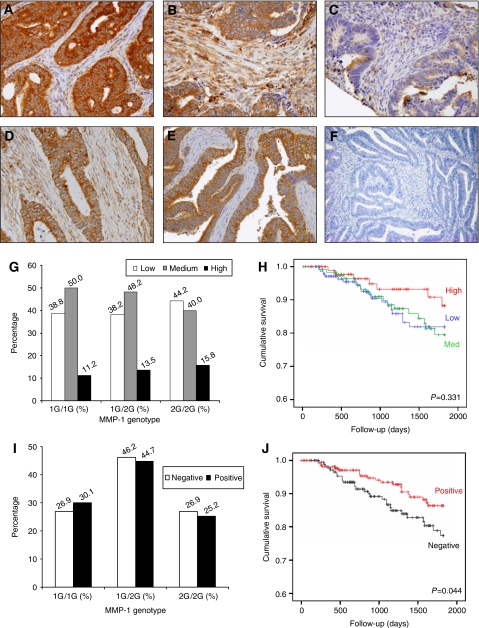
Immunohistochemical and survival analysis of MMP-1 from tissue microarrays. Immunohistochemical staining for MMP-1 in the epithelial tumour component demonstrated (**A**) high, (**B**) medium, and (**C**) low-level expression, whereas MMP-1 protein was either (**D**) present or (**E**) absent in the stromal component, (**F)** negative control incubated with no primary antibody. Distribution of MMP-1 expression as assessed by TMA **G**: MMP-1 tumour cell expression levels (low – white bars, medium – grey bars, high – black bars) was expressed as a percentage of the total number of patients in each genotype group. No significant difference was noted (*P*=0.633) in the distribution of MMP-1 protein expression as determined by TMA within each genotype group (**H**) Survival curves for MMP-1 expression within the epithelial cells of tumour tissue. No significant difference (*P*=0.331) was noted between the different MMP-1 protein levels. (**I**) Stromal MMP-1 expression (negative – white bars, positive – black bars) expressed as a percentage of the total number of patients in each group. There was no significant deviation (*P*=0.778) in the distribution of MMP-1 genotype based on stromal MMP-1 expression levels, (**J**) Survival curves for stromal MMP-1 expression. Positive immunostaining for stromal MMP-1 was associated with significantly better survival (*P*=0.044) compared to negative reactivity for stromal MMP-1.

Three distinct patterns of MMP-1 expression (high, medium, and low) were apparent in the tumour epithelium (*n*=363; Figure 4A–C respectively), whereas two patterns of MMP-1 expression (positive and negative) were displayed by the stromal component (*n*=402; Figure 4D and E, respectively). No significant association was found between MMP-1 genotype and MMP-1 protein expression either in the tumour (Figure 4G) or in the stromal component (Figure 4I). Interestingly, the presence of MMP-1 within tumour stroma was associated with increased overall survival (log rank test, *P*=0.044, Figure 4J), however no significant survival advantage was observed when MMP-1 protein was assessed in the tumour (log rank test, *P*=0.331, Figure 4H). Although this data is independent of MMP-1 genotype, it supports the notion that MMP-1 overexpression is indeed associated with favourable prognosis in our patient cohort.

### Loss of heterozygozity

Loss of heterozygosity (LOH) has been described at the MMP-1 -1607/2G polymorphic site in malignant melanoma (Noll *et al*, 2001). Our results showed no obvious LOH between the gDNA compared to the tumour-derived DNA in 100% of the randomly selected samples (*n*=61; data not shown) and this was comparable to other studies in renal (Hirata *et al*, 2003) and endometrial (Nishioka *et al*, 2000) carcinomas.

## DISCUSSION

In this report, we describe a PCR-based allelic discrimination assay for the MMP-1, -2, and -3 gene promoter polymorphisms. This is an accurate and reproducible screening tool, and along with an improved throughput capacity compared to previously published methods, may be the technique of choice for the rapid screening of large numbers of DNA samples. Although previous studies have documented a strong association between the 2G polymorphism in the MMP-1 promoter and the occurrence of several common cancers, including CRC, we were unable to corroborate these findings. At the same time, by using the largest patient/control cohort to date, and a robust and reliable PCR-based assay, we have shown that the 2G polymorphism in the MMP-1 promoter was a favourable prognostic indicator in patients with CRC as measured by a 5-year survival.

Several reports have demonstrated an association between MMP overexpression and poor prognosis in patients with cancer. For example, shorter disease-free survival was noted in patients with oral squamous cell carcinoma (SCC) expressing higher MMP-2 enzymatic activity (Yorioka *et al*, 2002). Matrix metalloproteinase-1 reactivity was found to be associated with poor outcome in patients with oesophageal (Murray *et al*, 1998) and CRC (Murray *et al*, 1996), whereas MMP-9 immunoreactivity correlated with a markedly poorer outcome in patients with SCC of the head and neck (5-year cause specific survival of 45% in MMP-9-positive tumours *vs* 92% in MMP-9 negative cases) (Ruokolainen *et al*, 2004). Matrix metalloproteinase-7 positively correlated with depth of invasion, lymph node metastasis, lymphatic invasion, tumour stage, and poorer outcome in patients with CRC (Adachi *et al*, 2001). Similarly, abundant MMP-11 (Chenard *et al*, 1996) and MMP-2 (Talvensaari-Mattila *et al*, 2003) immunoreactivity in tissue from patients with breast cancer corresponded to a shorter disease-free survival and poorer overall survival.

Despite this literature linking enhanced MMP production with poorer outcome for patients with cancer, the majority of these studies have been at best semi-quantitative and are a measure of expression at a single time point over the course of malignant progression (Chenard *et al*, 1996; Murray *et al*, 1996, 1998; Adachi *et al*, 2001; Papadopoulou *et al*, 2001; Yorioka *et al*, 2002; Talvensaari-Mattila *et al*, 2003; Roeb *et al*, 2004; Ruokolainen *et al*, 2004). The discovery of polymorphisms in MMP promoters that alter gene expression has revealed strong associations between these genetic variants and increased susceptibility to the development of malignancies and other diseases (Henney *et al*, 2000; Ye, 2000). The most extensively studied MMP SNP in relation to cancer is the −1607 bp insertion/deletion in the MMP-1 promoter (Rutter *et al*, 1998; Nishioka *et al*, 2000; Ghilardi *et al*, 2001; Zhu *et al*, 2001; Hinoda *et al*, 2002; Tower *et al*, 2002; Wyatt *et al*, 2002; Zinzindohoue *et al*, 2005; Elander *et al*, 2006). Similar studies have been conducted for the MMP-2 (Miao *et al*, 2003) and MMP-3 (Ghilardi *et al*, 2002; Hinoda *et al*, 2002) gene promoter polymorphism in relation to disease susceptibility and metastatic behaviour. These data suggest that functional polymorphisms, particularly those that influence high MMP expression, may act as susceptibility factors for cancer.

Despite the large number of studies that have found clear associations between MMP SNPs and susceptibility to malignant disease, some recent reports do not support these findings. Using a large cohort of patients with breast cancer, Krippl and colleagues (Krippl *et al*, 2004) demonstrated that the MMP-3 promoter polymorphism did not influence disease susceptibility. These results were subsequently confirmed in a smaller study using a different technique to establish subject genotype (Lei *et al*, 2002). The results of our investigation are consistent with these findings, in that we also were unable to demonstrate differences in the frequency of the MMP-1, -2, or -3 promoter SNP genotype between control subjects and patients with CRC. These findings suggest that analysis of these particular polymorphisms may not be useful indicators of the risk of developing cancer. As in the current investigation, other studies have found no significant differences in allele frequency for the MMP-1 promoter SNP in patients with colorectal (Biondi *et al*, 2000) or ovarian (Wenham *et al*, 2003) cancer compared to controls. These discordant results may be a consequence of the accuracy of the different techniques used to sequence these polymorphic sites, the source of DNA (tumour-derived compared to normal genomic), the number of subjects or the ethnicity of the population (Lai *et al*, 2005).

Despite the absence of a direct correlation between the MMP-1 2G polymorphism (associated with high MMP-1 protein expression) and MMP-1 staining in the tumour tissue, this comes as no surprise as MMP-1 tissue expression may be influenced by host immune response, disease stage, therapy, etc. Moreover, the immunohistochemical technique we employed may not be the method of choice to identify such an association, as it represents only a single period in tumour evolution. Our TMA results also highlight the potentially important contribution of MMP-1 production by stromal cells and indicate that tumour/stromal interactions may play a critical role in modulating gene expression to effect prognosis in patients with CRC (Kim *et al*, 2005). In the context of MMP function related to tumour progression, it has been speculated that enhanced MMP expression facilitates tumour cell invasion and metastasis, ultimately resulting in poor prognosis. Interestingly, as well as cancer promoting properties, cancer-inhibiting attributes have been recently documented for several MMPs. Indeed increased MMP-9 expression has been shown to favour survival in node-negative patients with breast cancer (Scorilas *et al*, 2001). Similarly, overexpression of MMP-1 in oesophageal carcinoma (Yamashita *et al*, 2001) and MMP-12 in patients with CRC (Yang *et al*, 2001) closely correlated with better prognosis, whereas the occurrence of elevated levels of a 50-kDa gelatinolytic MMP species (likely to represent MMP-1) in patients with breast cancer corresponded to better survival (Remacle *et al*, 1998). Although these findings contradict our current understanding on the cancer-promoting activity of MMPs, these enzymes have substrates other than typical structural matrix proteins. For example, it has been suggested that the mechanism by which endothelial cell proliferation and tumour angiogenesis is inhibited is via the ability of MMP-7, -9, and -12 to convert plasminogen in to angiostatin (Dong *et al*, 1997). This concept was further supported by studies that demonstrated the ability of MMP-9 to generate tumstatin (a potent angiogenesis inhibitor) from type IV collagen (Hamano *et al*, 2003) and the MMP-mediated formation of endostatin (Nilsson and Dabrosin, 2006). A beneficial role for MMP-1 has also been observed in atherosclerosis, whereby the active digestion of interstitial fibrillar collagen (types I, II, and III) by this enzyme affected cell differentiation and impaired cell migration (Lemaitre *et al*, 2001). A second mechanism, by which MMPs might function as inhibitors of tumour progression, is via their ability to cleave cell-surface receptors. The proteolytic cleavage of the oestrogen receptor *β* by MMP-26 was recently demonstrated by Savinov *et al* (2006) and this correlated with longer survival in patients with breast cancer. Finally, increased MMP activity has been shown to disrupt or denature adhesion molecules such as E-cadherin, thereby instigating tumour regression (Simian *et al*, 2006).

Confirmation of the protective effects of MMPs in cancer has come from animal experiments where tumours that arise from MMP-9 knockout (KO) mice were of a higher grade and displayed increased aggressiveness (Coussens *et al*, 2000). Likewise, squamous cell carcinoma induced in MMP-3 null mice grew more rapidly than tumours in control animals (McCawley *et al*, 2004). The authors proposed that MMP-3 plays a role in host defense during tumorigenesis, a likely mechanism, given the impaired immunity to bacterial infection in similar KO animals (Li *et al*, 2004). Increased incidence of skin tumours has been observed in MMP-8-deficient mice. Interestingly, protection against tumour development was restored when MMP-8-containing bone marrow cells were transferred back into these animals (Balbin *et al*, 2003). Other examples of the protective effect against tumour formation have been observed *in vivo* after treatment with synthetic MMP inhibitors (Coussens *et al*, 2002). Apart from the documented musculoskeletal pain and inflammation these agents cause in humans, there is evidence to suggest that they promote metastasis (Kruger *et al*, 2001).

To our knowledge, this is the first report that supports the hypothesis that a genetic variant in the MMP-1 promoter (associated with enzyme overexpression) favours overall survival in patients with CRC. Although the reason for this is unclear, host and/or tumour-related mechanisms are likely. A limitation of our study was that we did not confirm whether this polymorphism was indeed functional. There is, however, ample supportive evidence establishing the functionality of this polymorphism in both normal stromal cells (Wyatt *et al*, 2002) and tumour cells, including melanoma (Tower *et al*, 2003a) and breast cancer cells (Tower *et al*, 2003b). These studies confirmed that cells containing a 2G allele displayed enhanced transcriptional activity compared to cells harbouring the 1G allele. It was also noted that if MMP-1 transcriptional activity was not altered as a consequence of the MMP-1 polymorphism, then expression of this enzyme was likely to be differentially induced in response to cytokines and growth factors (Rutter *et al*, 1998; Tower *et al*, 2002; Wyatt *et al*, 2002). Investigations are currently underway in our laboratory to address this issue and to determine whether our observations are tumour-specific, or applicable to other adenocarcinomas such as breast and prostate. Finally, although our results may provide patients and physicians with a more accurate estimation of prognosis compared to methods currently available, they may also influence the timing and choice of chemotherapy for patients suffering from CRC.

## Figures and Tables

**Table 1:** Demographic characteristics and distribution of the MMP-1, MMP-2, and MMP-3 SNPs in CRC patients

**Groups**	**Patients *n* (%)**
*Sex*	
Male	265 (52.7)
Female	238 (47.3)
	
*Tumour stage*	
I	36 (7.2)
II	94 (18.7)
III	286 (58.9)
IV	87 (17.3)
	
*Average age (years)*	
Male	66.9
Female	70.5
Mean age in years (s.d.)	68.6 (12.1)
	
*MMP-1 SNP allelotype*	
1G	500 (51.0)
2G	480 (49.0)
	
*MMP-2 SNP allelotype*	
C	735 (75.6)
T	237 (24.4)
	
*MMP-3 SNP allelotype*	
5A	443 (47.1)
6A	497 (52.9)

Abbreviations: CRC, colorectal carcinoma; MMP, matrix metalloproteinase; SNP, single nucleotide polymorphisms.

**Table 2:** Patient data and clinicopathological characteristics of tumours according to MMP-1, -2, or -3 promoter SNP genotype

		**MMP-1**		**MMP-2**		**MMP-3**
		**High (2G/2G)**		**High (C/C)**		**High (5A/5A)**
		***vs***		***vs***		***vs***
	***n* (%)^a^**	**Low (1G/2G + 1G/1G)**	***n* (%)^a^**	**Low (C/T + T/T)**	***n* (%)^a^**	**Low (5A/6A + 6A/6A)**
*Sex*	490 (100)	0.43	486 (100)	0.06	470 (100)	0.22
Male	257 (52.4)		257 (52.9)		248 (52.8)	
Female	233 (47.6)		229 (47.1)		222 (47.2)	
*Tumuor location*	488 (99.6)	0.49	484 (99.6)	0.67	468 (99.6)	0.37
Left sided	308 (62.8)		309 (63.6)		301 (64.1)	
Right sided	180 (36.8)		175 (36.0)		167 (35.5)	
*Margin type*	474 (96.7)	0.54	472 (97.1)	0.13	459 (97.7)	0.86
Circumscribed	365 (74.5)		365 (75.1)		355 (75.5)	
Infiltrative	109 (22.2)		107 (22.0)		104 (22.2)	
*Grade*	490 (100)	0.71	486 (100)	0.81	470 (100)	0.46
High	66 (13.5)		64 (13.2)		60 (12.8)	
Low	424 (86.5)		422 (86.8)		410 (87.2)	
*Tumuor stage*	490 (100)	0.81	486 (100)	0.61	470 (100)	0.35
I	35 (7.1)		35 (7.2)		32 (6.8)	
II	91 (18.6)		91 (18.7)		86 (18.3)	
III	278 (56.7)		277 (57.0)		271 (57.7)	
IV	86 (17.6)		83 (17.1)		81 (17.2)	
*Lymph nodes mets*	490 (100)	0.20	486 (100)	0.89	470 (100)	0.57
Absent	310 (63.3)		306 (63.0)		300 (63.8)	
Present	180 (36.7)		180 (37.0)		170 (36.2)	
*Mucinous phenotype*	489 (99.8)	0.24	485 (99.8)	0.49	469 (99.8)	0.77
Absent	104 (21.2)		106 (21.8)		100 (21.3)	
Present	385 (78.6)		379 (78.0)		369 (78.5)	
*Lymphatic invasion*	479 (97.7)	0.78	475 (97.7)	0.95	460 (97.9)	0.91
Absent	373 (76.1)		368 (75.7)		356 (75.7)	
Present	106 (21.6)		107 (22.0)		104 (22.2)	
*Vascular invasion*	471 (96.1)	0.55	467 (96.1)	0.16	452 (96.2)	0.99
Absent	381 (77.8)		377 (77.6)		370 (78.7)	
Present	90 (18.3)		90 (18.5)		82 (17.5)	
*Intra-epithelial lymphocytes*	488 (99.6)	0.46	484 (99.6)	0.65	468 (99.6)	0.87
Inconspicuous	384 (78.4)		380 (78.2)		366 (77.9)	
Prominent	104 (21.2)		104 (21.4)		102 (21.7)	

a Percentage expressed as total of genotyped patients within each SNP group studied.

**Table 3:** Association of tumour characteristics and the MMP-1, MMP-2 and MMP-3 SNPs with CRC-associated death

	**OR**	**95% CI**	***P***
Tumour stage	5.96	4.20–8.50	<0.01
Vascular invasion	0.36	0.23–0.60	<0.01
Margin type	0.67	0.43–1.05	0.08
			
*MMP-1*			
2G/2G *vs* 1G/2G+1G/1G	0.43	0.19–0.96	0.04
1G/1G *vs* 1G/2G+2G/2G	1.72	0.95–3.14	0.08
			
*MMP-2*			
C/C *vs* C/T+T/T	1.16	0.66–2.04	0.60
T/T *vs* C/T+C/C	0.67	0.16–2.74	0.57
			
*MMP-3*			
5A/5A *vs* 5A/6A+6A/6A	1.59	0.87–2.84	0.12
6A/6A *vs* 5A/6A+5A/5A	1.17	0.63–2.17	0.62
